# Treelink: data integration, clustering and visualization of phylogenetic trees

**DOI:** 10.1186/s12859-015-0860-1

**Published:** 2015-12-29

**Authors:** Christian Allende, Erik Sohn, Cedric Little

**Affiliations:** Faculty of Engineering and Sciences, Universidad Adolfo Ibañez, Diagonal las Torres 2640, Santiago, 7941169 Chile

**Keywords:** Phylogenetic tree, Data integration, Clustering, Visualization

## Abstract

**Background:**

Phylogenetic trees are central to a wide range of biological studies. In many of these studies, tree nodes need to be associated with a variety of attributes. For example, in studies concerned with viral relationships, tree nodes are associated with epidemiological information, such as location, age and subtype. Gene trees used in comparative genomics are usually linked with taxonomic information, such as functional annotations and events. A wide variety of tree visualization and annotation tools have been developed in the past, however none of them are intended for an integrative and comparative analysis.

**Results:**

Treelink is a platform-independent software for linking datasets and sequence files to phylogenetic trees. The application allows an automated integration of datasets to trees for operations such as classifying a tree based on a field or showing the distribution of selected data attributes in branches and leafs. Genomic and proteonomic sequences can also be linked to the tree and extracted from internal and external nodes. A novel clustering algorithm to simplify trees and display the most divergent clades was also developed, where validation can be achieved using the data integration and classification function. Integrated geographical information allows ancestral character reconstruction for phylogeographic plotting based on parsimony and likelihood algorithms.

**Conclusion:**

Our software can successfully integrate phylogenetic trees with different data sources, and perform operations to differentiate and visualize those differences within a tree. File support includes the most popular formats such as newick and csv. Exporting visualizations as images, cluster outputs and genomic sequences is supported. Treelink is available as a web and desktop application at http://www.treelinkapp.com.

**Electronic supplementary material:**

The online version of this article (doi:10.1186/s12859-015-0860-1) contains supplementary material, which is available to authorized users.

## Background

Phylogenetic trees are increasingly used to visualize comparative information in an evolutionary context [1], e.g. comparing strands of viruses and genes within different clades to find new ways of classifying and differentiating groups of leafs within a tree. A wide variety of visualization and annotation tools have been developed in the past [2–4] however none of them are intended for integrative and comparative analysis.

Information that can be interpreted in a phylogenetic context is growing rapidly, creating a continuous need to find new ways to integrate, process and deliver this new information. Furthermore, there is also a demand to explore, compare, display and interpret trees using information not directly contained in these trees, such as taxonomy, geography, and traits, among others [5].

Treelink was designed with those needs in mind, enabling an easy and automated procedure that links data sources to nodes and topologies, which in turn allow the construction of comparative representations that can be explored and extracted.

Representation of large phylogenies and clustering of nodes has also proven to be difficult in epidemiology and evolutionary research [6], where large and complex trees are used for exploration and pattern analysis. A novel clustering algorithm was developed that groups and divides clades and sequences within a tree topology based on their divergence measure, resulting in characteristic and representative sets that simplify phylogenies and reveal similarity.

## Implementation

The main design directives behind Treelink were: (i) Ease of use (ii) Automation (iii) Fast Performance (iv) Scalability (v) Aesthetics.

The application’s first step is to provide automated data integration and visualization for additional renderings of the tree. The main features include:

### Data integration

Data integration from standard datasets is executed by linking the leaf label to a key in the dataset. Once the integration process is finished, the aggregated information is displayed by hovering over the nodes. The corresponding table can also be shown for additional operations.

### Classification and cross-reference

**Fig. 1:**
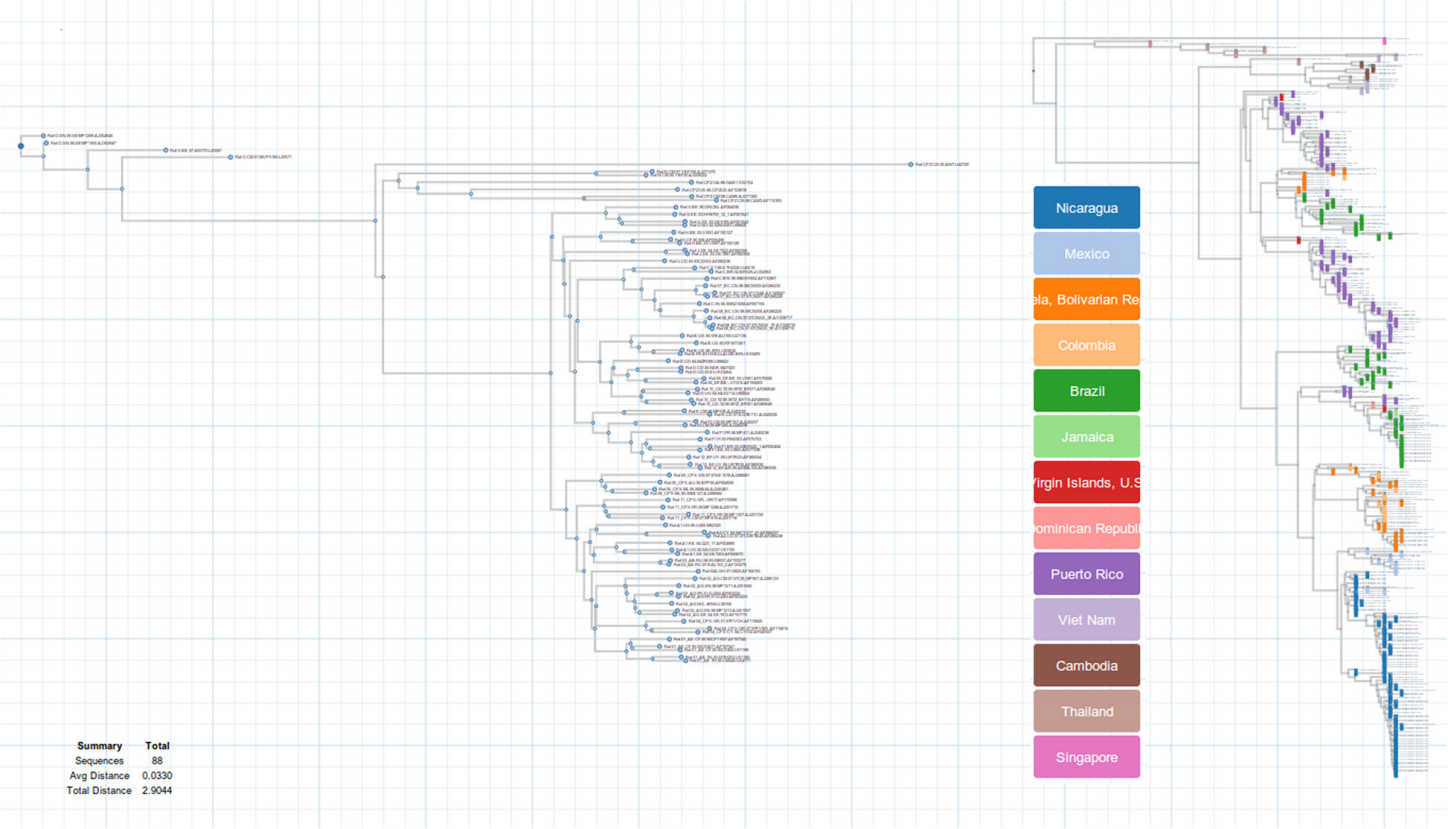
Phylogenetic visualizations. Left: HIV Subtype Consensus tree. Right: HIV Consensus tree classified by the country field of a dataset

The application comes with 2 general purpose functions based on data integration. The user can visualize and cross-reference specific attributes from the dataset by searching for them inside the leafs or by selecting them on the table. An annotated visualization is then rendered by the software showing the distribution of those fields on the tree (Fig. 1). Classification is accomplished by selecting a categorical trait or property loaded from the dataset, which is distributed and displayed along the leafs of tree.

### Sequence extraction and visualization

Sequence information in FASTA format can also be linked to the selected phylogeny for extraction and visualization purposes. By selecting leafs and internal nodes, the user can visualize or export the subset of genomic or proteonomic sequences into a new file.

### Clustering and tree simplification

Treelink also includes a clustering and tree simplification component called TreeClus, based on a novel algorithm that divides and clusters phylogenetic topologies based on a dissimilarity measure calculated from the evolutionary distance of the branches (for more details, (see Additional file 1)). This function can be used for detecting characteristic subgroups within a given topology for differential analysis in evolution, e.g. discerning subtypes in viral phylogenies [7] and classifying taxa.

### Ancestral reconstruction and phylogeography

Phylogeographic inference from tree topologies and datasets is implemented in the TreeMap component, through ancestral character state reconstruction [8] for different evolutionary models: Linear parsimony with a delayed and accelerated transformation rule, and a likelihood approach [9], for cases assuming some version of Brownian motion. The resulting tree and states can then be plotted on a map, where the movement of states is animated proportional to evolutionary time.

## Results

### Output

The main outputs of Treelink comprise of browser viewable and downloadable svg graphics for the following results:

- Visualizations of attribute searches or a classification by a field
- FASTA files from a subtree or leaf
- Tabulated files of the sequences and their cluster
- Clustered Tree Visualizations
- Ancestral Reconstruction Visualizations
- Phylogeography Visualizations

### Software comparison

A major point of difference between Treelink and alternative software (e.g., iTol, TreeDyn) [2–4, 10] is its automated data integration process, that doesn’t require formatting or pre-processing on datasets to fit input requirements. Other existing tools require manual annotation of meta-data to associate or attach information to selected tree elements. Treelink overcomes these requirements by using standard dataset formats as an integration source, relieving the user of tasks like manual annotations on the leafs and permits integrating associated data directly from the sources of data collection, given that csv is a popular export format of sql-based databases, excel and other spreadsheets.

Another upside includes the amount of fields that can be linked to the tree, allowing up to 9 different fields to be integrated. Performance and flexibility distinctions include the ability to navigate and interact with the tree by selecting subtrees and creating diverse visualizations almost instantaneously after the initial data integration step with the same data source.

The design based on simplicity requires little training or knowledge of the tool, labels are annotated automatically with corresponding legends and attributes can be search directly into the tree. An short interactive tutorial found on the site gives a quick overview of the main functions and use of the tool. A short manual is also included for additional knowledge on the limitations and capabilities of Treelink.

The development on the javascript D3 library proves to be an additional benefit, allowing a more interactive visualization and navigation of large trees, it also enables to use its diverse visualization capabilities and its data-driven approach to DOM manipulation. The additional sub-components such as clustering, ancestral reconstruction and phylogeographic mapping exhibit some of the applications that can be built on top of Treelink and the D3 library.

### Worked examples

**Fig. 2:**
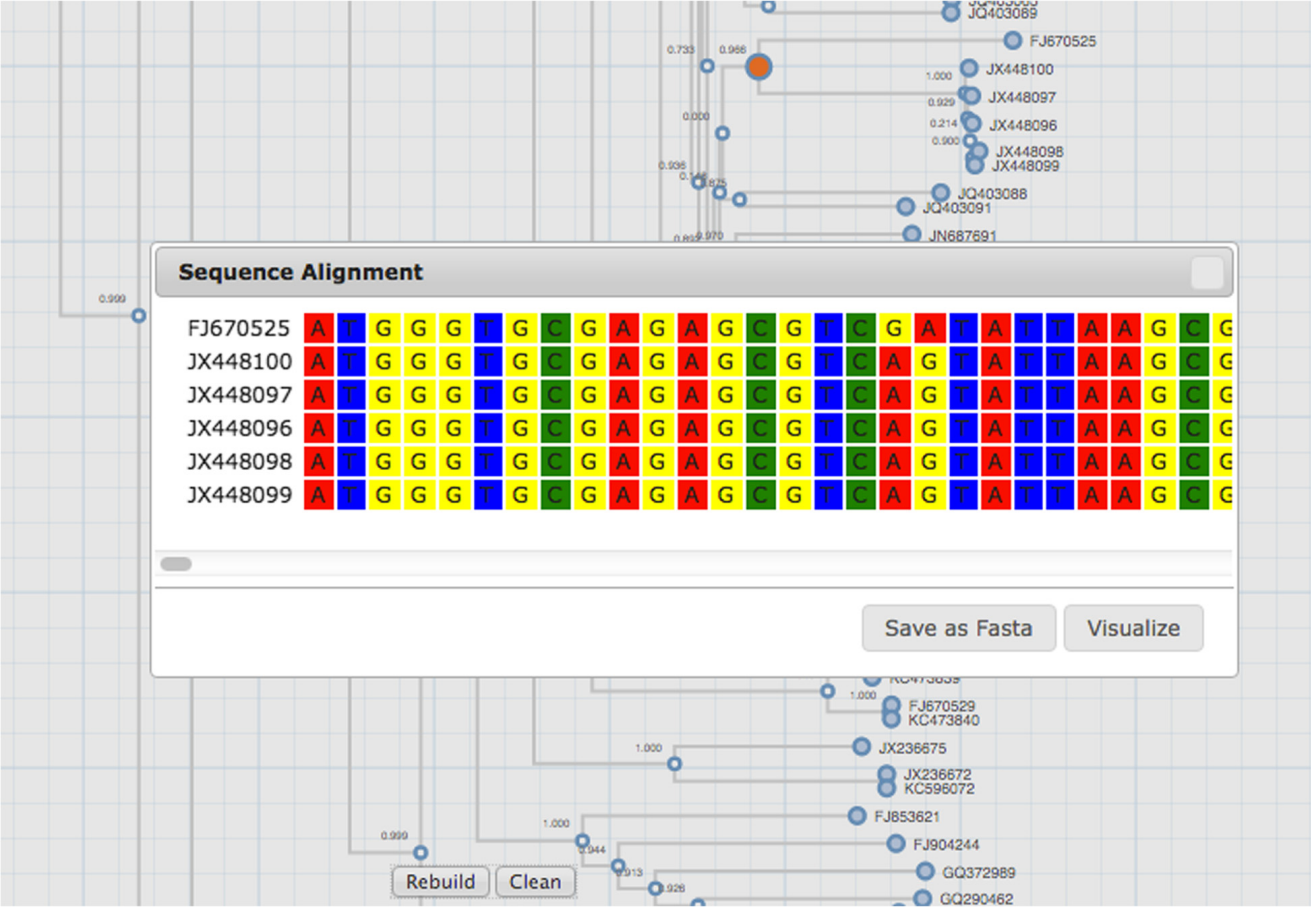
Sequence Extraction. Sequence visualization of an internal node selection

**Sequence extraction** Data integration of fasta files and phylogenetic trees can be useful for selection and extraction of sequences of interest. By selecting an internal node or individual leafs, sequence ids are stored in internal variables that can be later used to link, visualize or extract a list of genomic or proteonomic sequences (Fig. 2).

**Fig. 3:**
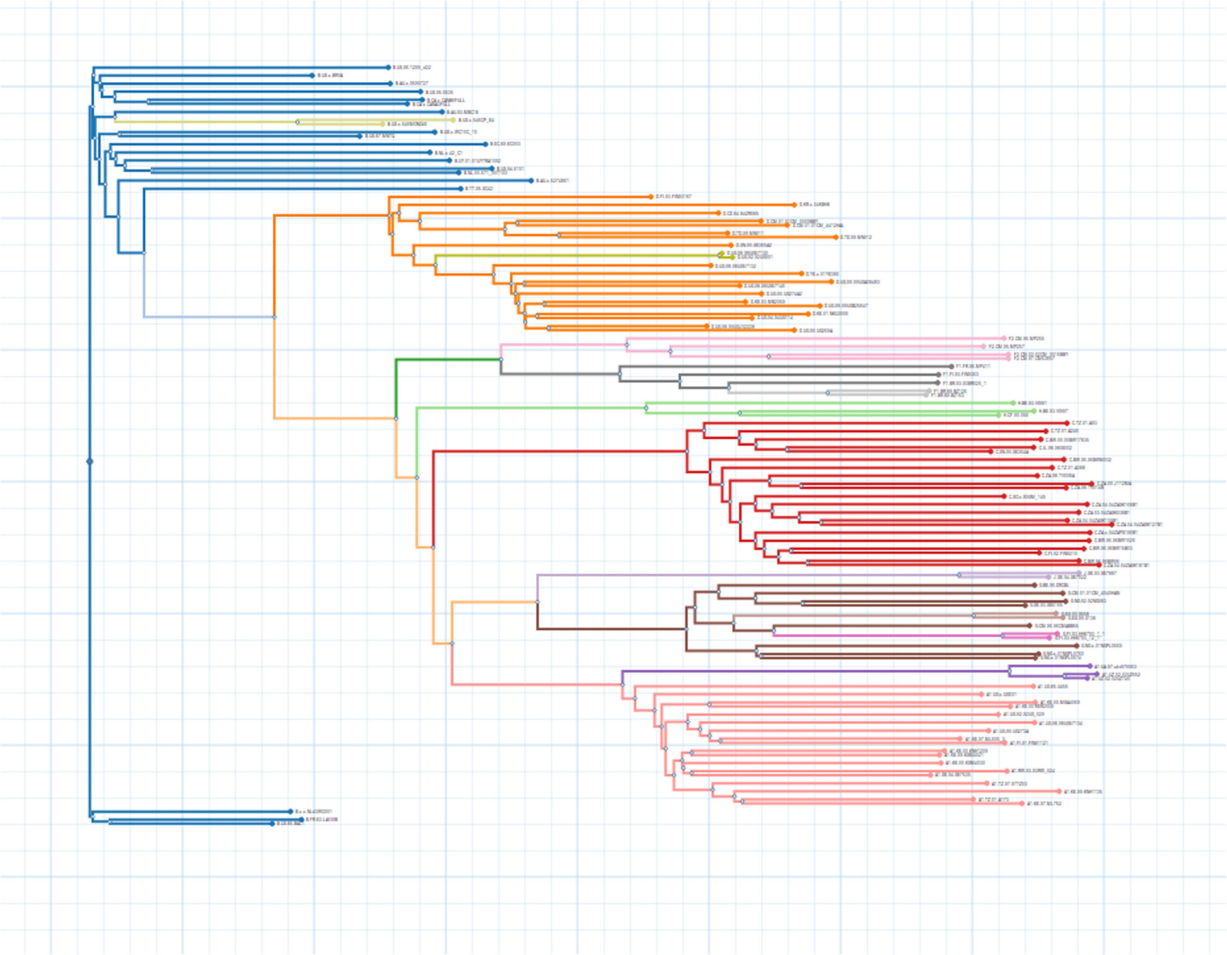
Clustering. Phylogenetic clustering of european HIV-1 subtypes

**HIV subtyping** HIV gene sequencing and phylogenetic inference is used to identify and classify HIV-1 subtypes [7], and phylogenetic clustering has proven to be a powerful tool to understand the forces that shape patterns of viral sequence diversity [11]. In the following example the clustering algorithm is executed on an HIV-1 tree resulting in a divided and clustered representation that reveals the different subtypes it holds (Fig. 3).

**Fig. 4:**
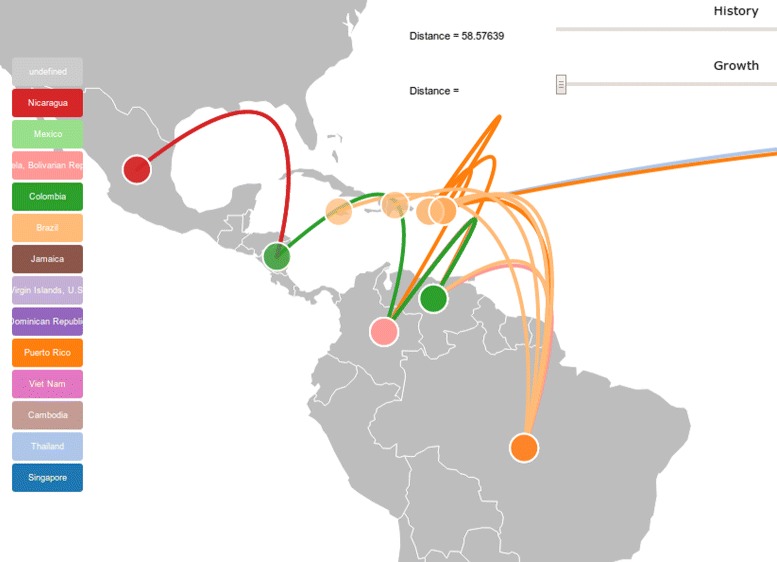
Phylogeographic plotting. HIV subtype B consensus tree plotted in Central and South America

**Phylogeography** Phylogeography studies the historical processes that could be responsible for geographic distributions of individuals [12]. In this example an HIV subtype B consensus tree was linked to a dataset that contained the countries of each sequence. Ancestral character reconstruction was performed by a maximum parsimony algorithm [13] that assigned states for the ancestral nodes. Then the result was linked and plotted on a geographical map to visualize the movement of country states in accordance to the tree structure (Fig. 4).

## Conclusion

Treelink is a comprehensive open-source solution for rendering and integrating datasets into phylogenetic trees. It supports all operating systems and has an HTML5 version for the widest possible audience. File support includes the most popular formats like newick and csv, generating interactive trees that incorporate analytics in scalable vector images. It can also be used as a library to extend and complement with additional functions and methods, and can be easily integrated into existing web applications.

## Availability and requirements

**Project name:** Treelink**Project Website:** http://www.treelinkapp.com**Project Github:** https://github.com/allendecid/TreeLink**Programming language:** Javascript**Libraries:** D3, jquery**Requirements:** A modern browser**License:** MIT

## Additional file

Additional file 1
**Clustering Algorithm.** The clustering algorithm, along with the mathematical explanation based on graph theory. Pseudo-Code included. (PDF 169 kb)
