# Supplementary material for: Treelink: data integration, clustering and visualization of phylogenetic trees
Source: BMC Bioinformatics. 2015 Dec 29;16:414. doi: 10.1186/s12859-015-0860-1 (PMC4696249; doi:10.1186/s12859-015-0860-1)
Supplement: Additional file 1 — Clustering Algorithm. The clustering algorithm, along with the mathematical explanation based on graph theory. Pseudo-Code included. (PDF 169 kb) [file 12859_2015_860_MOESM1_ESM.pdf]

TreeLink Clustering Algorithm

The proposed algorithm uses the distribution of branchlengths (or evolutionary distance) to divide and cluster clades to define groups into statistically significant subsets of the tree.

The detection phase

Initially the algorithm detects the tree topology based on the diversification type:

Late diversification or early diversification, in the earlier case, the algorithm will include the leafs in the parameters, and in the later case it will not. A mixed model can be used in which case it will assess each clade and classify it for diversification type. Comparing the averages of the leaf distances with the internal node distances makes the detection.

The order phase

The algorithm navigates the tree saving all the node distances of the tree in an associative array with the id of the node that contains it. After the search is done it takes the evolutionary distance as the dissimilarity measure and orders all the individual evolutionary distances based on their dissimilarity magnitude from higher to lower.

Step1: After the ordering is done it searches for changes in dissimilarity gained in step processes, defined as the local minima of the ordered distribution of the distances, and will save them into another variable. The accumulated dissimilarity gained by selecting that cut value is also stored.

Step2: After the local optimums are saved with the accumulated dissimilarity, the selection of the optimum is done. Better selections of the optimum include the value of the optimum (or the change in dissimilarity) and the accumulated dissimilarity into the analysis.

Step3: A recursive function navigates the tree and assigns a cluster number to the nodes that fulfill the condition of having a larger evolutionary distance than the optimum selected.

The output phase

After the assignment to clusters is done, the tree hierarchical structure is flattened into and array of objects with the node names and the cluster number.

Mathematical Definitions

Definition. A graph G is pair G = (V, E) where V = V (G) is a set of vertices or nodes, and E = E(G) is a set of edges. Each edge e $\in$ E is a two-element set e = {v1, v2} of vertices v1, v2 $\in$ V .

Definition. A tree T = (V, E) is a connected graph with no cycles.

Definition. A metric tree (T, w) is a rooted or unrooted tree T together with a function w : E(T) → R ≥0 assigning non-negative numbers to edges. We call w(e) the length or weight of the edge e.

Definition. Edge Space E(G). Is the vector space freely generated by the edge set E. The dimension of the edge space is the number of edges or card(E).

Definition A Cut C= (S,T) is a partition of V of graph G=(V,E) into two subsets, S and T.

Dissimilarity measure:

Let W = { $w(e_1)$, $w(e_2)$, ..., $w(e_n)$ } be the collection of measurements that quantitatively separate two pairs of edges.

Then we note the total dissimilarity contained in the edges of the metric tree as the sum:

$$\text{Tdis}=\sum_{j=1}^{n} w(e_j)$$

Where n= card(T)

And the ordered set B of lengths of edges is defined as the sequence:

$B=\{b_1,b_{2,...,}b_n\}=\{w(e) \in T: w(e)_n \leq w(e)_{n-1} : n=1,2,....,\text{card}(T)\}$

And the dissimilarity explained by the number of edges as the partial sum of the sequence B:

$$\sum_{j=1}^{m} b_j / \text{Tdis}$$

Where m≤card(T).

Optimums

In order to explain most of the dissimilarity contained in the graph in a limited number of subsets, changes in dissimilarity explained by the current value in the ordered set of B are calculated.

where

$$x \in X : x = \underset{b \in B^o}{\text{argmin}}\, f(b)$$

Where X is the set of optimum values that cut the tree for a portion of dissimilarity explained with a given number of subsets of T.

Cuts C

$C=\{S_1,\ldots,S_n: w(e_{n-1}) \geq \mathbf{x} = \mathbf{argmin}\ \mathbf{f}(\mathbf{b})\ \}$

Where C is the set of clusters or subsets S for n edges in the Metric Tree T.

Pseudocode

Tree to array

```
Function flatten(tree)
Array[tree.node.id]=tree.node.branchlength
                for (i=0; i<tree.children.length;i++)
                flatten(tree.children[i])
                end for
end function
```

Sort Lengths

```
for(i=0; i<Array.lenght; i++)   Put the next smallest element in location A[i];
```

Optimum

```
function min(A)
for(i=0; i<A.lenght; i++)
        if(A[i]<A[i-1] and A[i]≅A[i+1])
        return A[i]
        end if
end for
end function
```

Make Cuts

```
Function cut(tree,optimum,array)
cluster = 0;
    Function navigatetree(subtree, optimum,cluster)
        if ( node.length >= optimum )
    cluster+=1
```

```
            array[subtree.node.id]=cluster;
                    for (i=0; i<subtree.children.length;i++)
                    navigatetree(subtree.children[i], optimum, cluster)
                    end for
            end if
            else
            array[subtree.node.id]=cluster;
                    for (i=0; i<subtree.children.length;i++)
                    navigatetree(subtree.children[i], optimum, cluster)
                    end for
            end else
        end function
navigatetree( tree, optimum, cluster);
return tree;
end function
```
